# Image of a thawed frozen specimen obtained using a cryoprobe floated with oil droplets in normal saline: An endobronchial lipomatous hamartoma image

**DOI:** 10.1002/rcr2.1318

**Published:** 2024-03-05

**Authors:** Yuki Takigawa, Ken Sato, Tomoyoshi Inoue, Masahiro Takada, Keiichi Fujiwara

**Affiliations:** ^1^ Department of Respiratory Medicine NHO Okayama Medical Center Okayama Japan

**Keywords:** cryoprobe, endobronchial lipomatous hamartoma, floating tumour, oil droplet, thawed frozen specimen

## Abstract

Hereby, we present a rare case of a resected endobronchial tumour that floated or showed oil droplets in saline. In this study, we report an interesting image related to endobronchial lipomatous hamartoma cryotherapy.

## Graphical abstract

We present an unusual and rare case of a resected endobronchial tumour floating or displaying oil droplets in saline. The mass was pathologically diagnosed as an endobronchial lipomatous hamartoma (ELH).

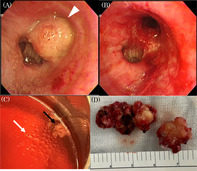

A man in his mid‐80s was referred to our hospital for endobronchial tumour resection. We applied rigid bronchoscopy under general anaesthesia and observed a smooth‐surfaced tumour without vascular growth in the left upper lobe (Figure 1A). Resection was not possible with a high‐frequency snare as the surface and polyp stalk was undetected. Thus, we performed cryotherapy using a 2.4‐mm cryoprobe. Initially, we performed the intervention with 3–5‐s freezing, followed by final freezing and tumour removal using 10‐s freezing (Figure 1B). After freezing, we thawed the obtained frozen specimen in normal saline. We observed that the specimen and oil droplets were floating (Figure 1C,D). The mass was pathologically diagnosed as an endobronchial lipomatous hamartoma (ELH), a relatively rare tumour (Figure 2). Certain studies described ELH resection using a high‐frequency snare. Recently, bronchoscopic treatment has tended to take place over surgery^1^ and some cases of resection under cryotherapy were documented.^2^ Since the density of fat is lower than that of normal saline, we expected ELH floating due to excessive fat. We considered the acquired image of interest as it presents an unusual and rare case of a resected endobronchial tumour floating or displaying oil droplets in saline.

**FIGURE 1:**
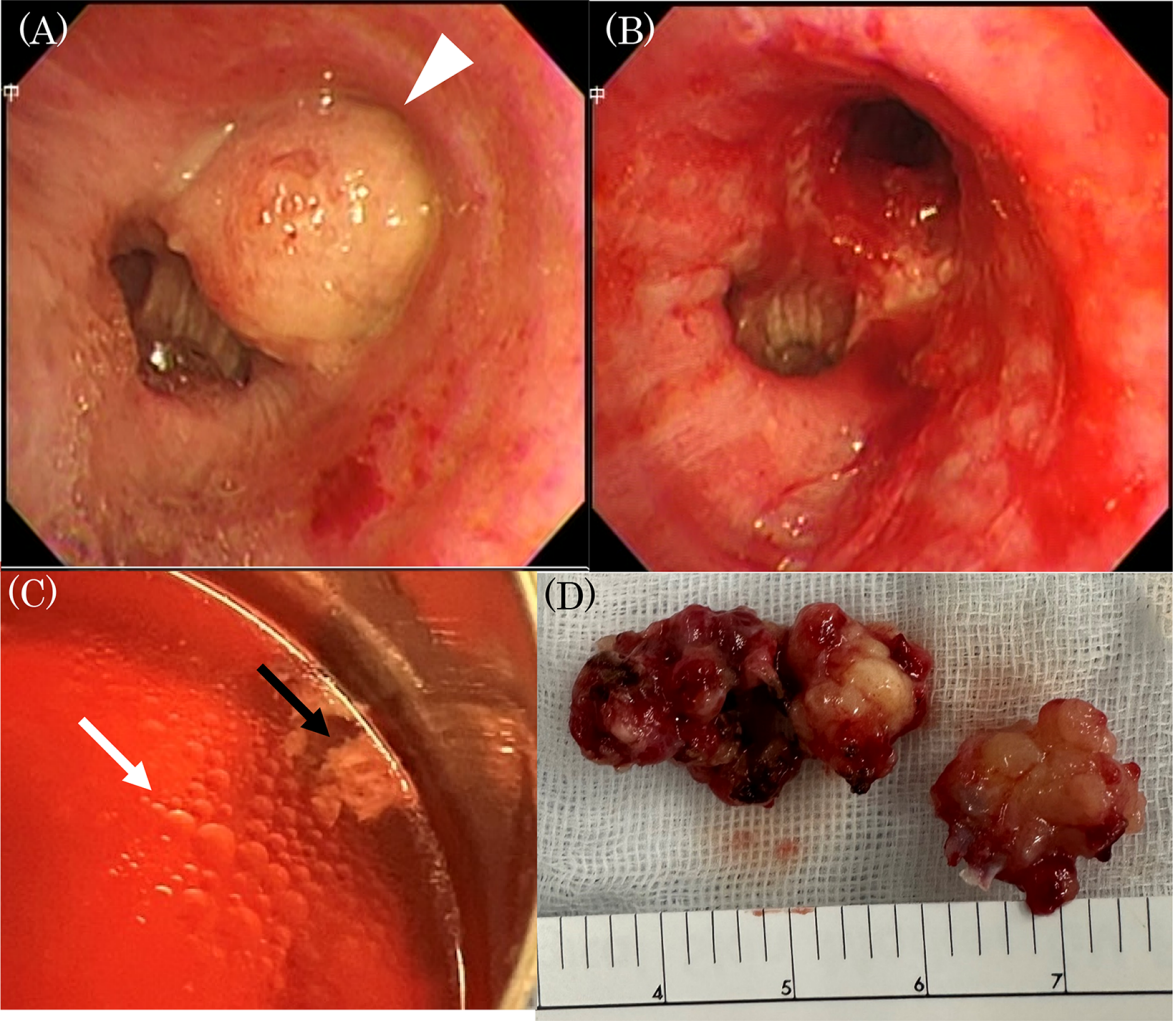
(A) Bronchoscopy highlighting a tumour obstructing the left upper lobe. (B) Intact airway patency of the left main bronchus after resection. (C) Specimen, obtained using a cryoprobe, (black arrow) and oil droplets (white arrow) floating in normal saline. (D) A collection of tumours resected using a cryoprobe. Specimen size: 4 cm × 2 cm.

**FIGURE 2:**
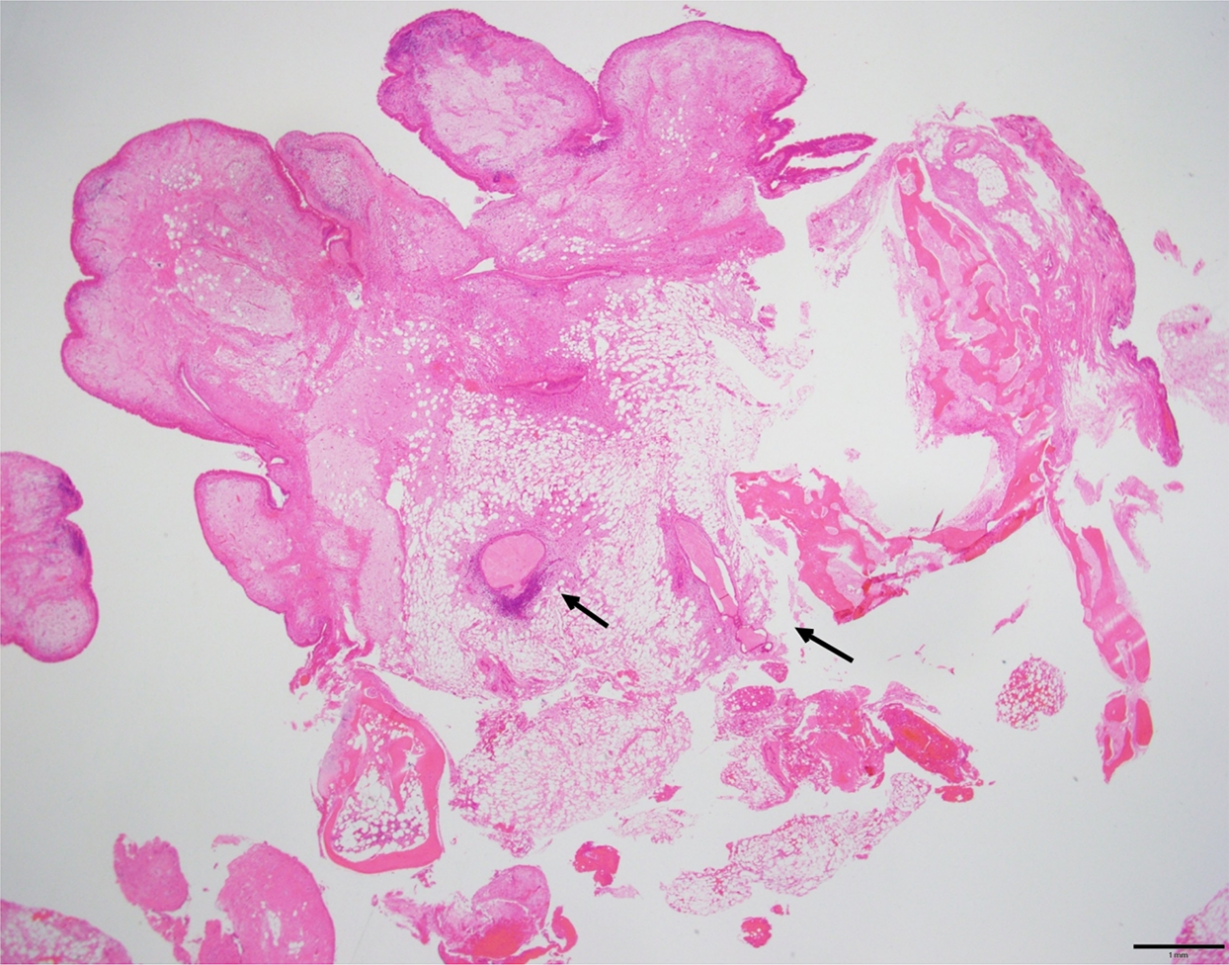
A histopathological image showing an abundance of mature fat cells and a few bronchial glands (arrows). These findings leads to the diagnosis of lipomatous hamartoma (×40).

## AUTHOR CONTRIBUTIONS

Yuki Takigawa and Ken Sato wrote the manuscript, which was then reviewed by all co‐authors.

## CONFLICT OF INTEREST STATEMENT

None declared.

## ETHICS STATEMENT

The authors declare that appropriate written informed consent was obtained for the publication of this manuscript and accompanying images.

## Data Availability

The data that support the findings of this study are available from the corresponding author upon reasonable request.
